# Supplementary material for: Mapping and DNA sequence characterisation of the Rysto locus conferring extreme virus resistance to potato cultivar ‘White Lady’
Source: PLoS One. 2020 Mar 31;15(3):e0224534. doi: 10.1371/journal.pone.0224534 (PMC7108733; doi:10.1371/journal.pone.0224534)
Supplement: S1 Table — (DOCX) [file pone.0224534.s001.docx]

**Supplementary Table 1**

**List of primer pairs used in this study**

| **Name** | **Sequences (5’→3’)** | **Used for** | **Reference** |
| --- | --- | --- | --- |
| STM0003 | GGAGAATCATAACAACCAG  AATTGTAACTCTGTGTGTGTG | mapping | Milbourne et al. (1998) |
| SGN-U256066 | AGCCTGGTGCTGTCGATGTGT  CTCTCATACGTTGGCATGCCG | mapping | This study |
| YES3-3A | TAACTCAAGCGGAATAACCC  AATTCACCTGTTTACATGCTTC | mapping and isolation of 365B5 | Song and Schwarzfischer (2008) |
| ST1 | atgcgggagaacacgatac  TGTGAGGCAATGTGGGATG | mapping and isolation of 154G1, 313F7, 161A6, 704E10, 109D9 | Decsi et al. (2012) |
| Cat-in2 | TGACAACAAATGCTGGTGGT  AAGGTGGCAAGCTTCTCAAT | mapping | Cernák et al. (2008a) |
| Sec15 | TCAACCAAGACTCCACGCAG  AAACATTGTGTTGGTCGGCAA | mapping | This study |
| CadInd | AGGGCAGATCAAGTTCCTTTCCTG  AAACATGCCACGTGGAAAGCTGAA | mapping | This study |
| 1.365 | TTCACAACTTGACCCTCGGA  TTTTGCTGAGCATAGGCAACT | mapping and isolation of 626B1, 326C1, 139F7 | This study |
| DisRes.spec | ACGCGCAAGAAGGACGTGTGT  AGCTCAACGCGAGGACACCAT | isolation of 156F6, 110A10; detection of the disease resistance gene in Désirée plants transformed with the DR construct | This study |
| 1.110 | TGCATGGGTGCATTACCTTA  CATTTGATGTTGACCCATGTTC | isolation of 164H4 | This study |
| 1.109 | CGACCCTCCAACACTTCTTC  ATACACCCAGCAGATTCGTCA | isolation of 443B9 | This study |
| 1.156 | ATCGTCTGCAAGCTCTTGGG  GCCCCGCATGCAATATGAAC | validation of 110A10, 626B1, 326C1, 139F7 | This study |
| Phloem1-2 | TGGATCCCTTATCCAGACGAGGTCAA  TGGATCCCGGAATAGTGAGGGAGGA | cloning of the phloem resistance genes 1 and 2 into pBIN19 | This study |
| Phloem 3-4 | TGGATCCTTATTTCCGTTTGTTTTGCC  TGGATCCTGTGCCTTTTATGTTTTCCT | cloning of the phloem resistance genes 3 and 4 into pBIN19 | This study |
| DisRes29344 | GCAGGATCGAGCCAAAAGTT  ACTCTAATAGAAGTGGCAACAAGA | cloning of the disease resistance gene into pBIN19 | This study |
| TMV2 | CGGATCCTGACGGAAGAAACAAATGAC  TGGATCCGCGTACACATCATTCGTTT | cloning of the TMV resistance gene 2 into pBIN19 | This study |
| TMV3 | TGGATCCTCCTCGTGAACATACTGG  CGGATCCAACTCCGTATTCAACCAA | cloning of the TMV resistance gene 3 into pBIN19 | This study |
| Phloem1.spec | CTTATCCAGACGAGGTCAA  CAAATGCATCCCTCCATCTT | detection of the phloem protein-coding gene 1 in Désirée plants transformed with the P1-2 construct | This study |
| Phloem4.spec | TGCATCGTCAAAATGGTGTT  TGTGCCTTTTATGTTTTCCT | detection of the phloem protein-coding gene 4 in Désirée plants transformed with the P3-4 construct | This study |
| TMV2.spec | GATGTCAAGGACGATAAACCTG  TAAGAGAAATGGGAGAAAATGTCA | detection the expression of *TMV2* in Désirée and S440 plants transformed with the T2 construct | This study |
| TMV3.spec | GCTTCCACTTACATCCAACTTTC  ACAGCTCGCTATGCTTTGTTT | detection of *TMV3* in Désirée and S440 plants transformed with the T3 construct | This study |
| PVY CP | AAGGATCCGCTTTCACTGAAATGATGG  AGGGAAGCTTCTAGAGTCTCCTGATTGAAG | detection of PVY^NTN^ in Désirée and S440 plants | Bukovinszki et al. (2007) |
| BAC154-3 Fw  BAC164-5 R | GCCTCTCACTTTCAGGCTATG  TGATGAACGAAGCACGATCTAC | cloning the adjacent end regions of 154G1 and 164H4 | This study |

*Remark: Bam*HI restriction sites used for cloning are underlined.

**References**

Bukovinszki A, Götz R, Johansen E, Maiss E, Balázs E (2007) The role of the coat protein region in symptom formation on *Physalis floridana* varies between PVY strains. Virus Res 127:122-125

Cernák I, Decsi K, Nagy S, Wolf I, Polgár Z, Gulyás G, Hirata Y, Taller J (2008) Development of a locus-specific marker and localization of the *Ry_sto_* gene based on linkage to a catalase gene on chromosome XII in the tetraploid potato genome. Breeding Sci 58:309-314

Decsi K, Cernák I, Bánfalvi Z, Korom E, Wolf I, Vaszily Z, Taller J, Polgár Z (2012) Marker assisted selection of the *Solanum stoloniferum* based PVY resistance in the breeding material of Keszthely. ScienceMED3:215-219

Milbourne D, Meyer RC, Collins AJ, Ramsay LD, Gebhardt C, Waugh R (1998) Isolation, characterization and mapping of simple sequence repeat loci in potato. Mol Gen Genet 259:233–245

Song YS, Schwarzfischer A (2008) Development of STS markers for selection of extreme resistance (*Ry_sto_*) to PVY and maternal pedigree analysis of extremely resistant cultivars. Amer J Potato Res 85:159–170
