# Supplementary material for: Mapping and DNA sequence characterisation of the Rysto locus conferring extreme virus resistance to potato cultivar ‘White Lady’
Source: PLoS One. 2020 Mar 31;15(3):e0224534. doi: 10.1371/journal.pone.0224534 (PMC7108733; doi:10.1371/journal.pone.0224534)
Supplement: S1 Fig — (DOCX) [file pone.0224534.s002.docx]

TGGAGTAACGGAGGCTGACCAAATCCCTGCAAGTCATAGAATGAAATCAGACCTC**ATGCGGGAGAACACGATAC**ATGATACTAAAAGATTAAATGTTTCTGAATGTTCCTTCTCTCCAGGTACATAACTATCCAGAAAGGAAACTGGAACTTATCAGTAAGTCATTTCAATAACCATTTCGTGGCACGACAATGAACTTACCCTTGGCCTGCCACAATCCCTCAAGTTGGCCTGCCCACAATCCCTCAAGTTTGTAATTCCCATCTTGATTTTACCAAATAAACAGTATTCCCTTGATCTACCAGCATTTTTCATCAAGATATAGAACA**CATCCCACATTGCCTCACA**CATAAAACAAATCTCAAGTTTACGGACGATGCTTTACAAGAAAAAACATGTTAAACAAACAAACCTGTATCCGTCCCTTTCTTGTTGCAATATGATGTGCTGTATTTCCTTTGCTATCTTCCAAGGCCAAAACAGCAGGATTTGGCTTGATTAATTCGAGCACGATGTCAACATTTTGTCCCTTTGCAGCCAGTGTA

**Fig. S1. Nucleotide sequence of the SCAR marker ST1 converted from a RAPD marker.** Sequence of the forward primer is in red, while the complementary sequence of the reverse primer used in PCR is in green.
