# Supplementary material for: Mapping and DNA sequence characterisation of the Rysto locus conferring extreme virus resistance to potato cultivar ‘White Lady’
Source: PLoS One. 2020 Mar 31;15(3):e0224534. doi: 10.1371/journal.pone.0224534 (PMC7108733; doi:10.1371/journal.pone.0224534)
Supplement: S3 Fig — (DOCX) [file pone.0224534.s004.docx]

**
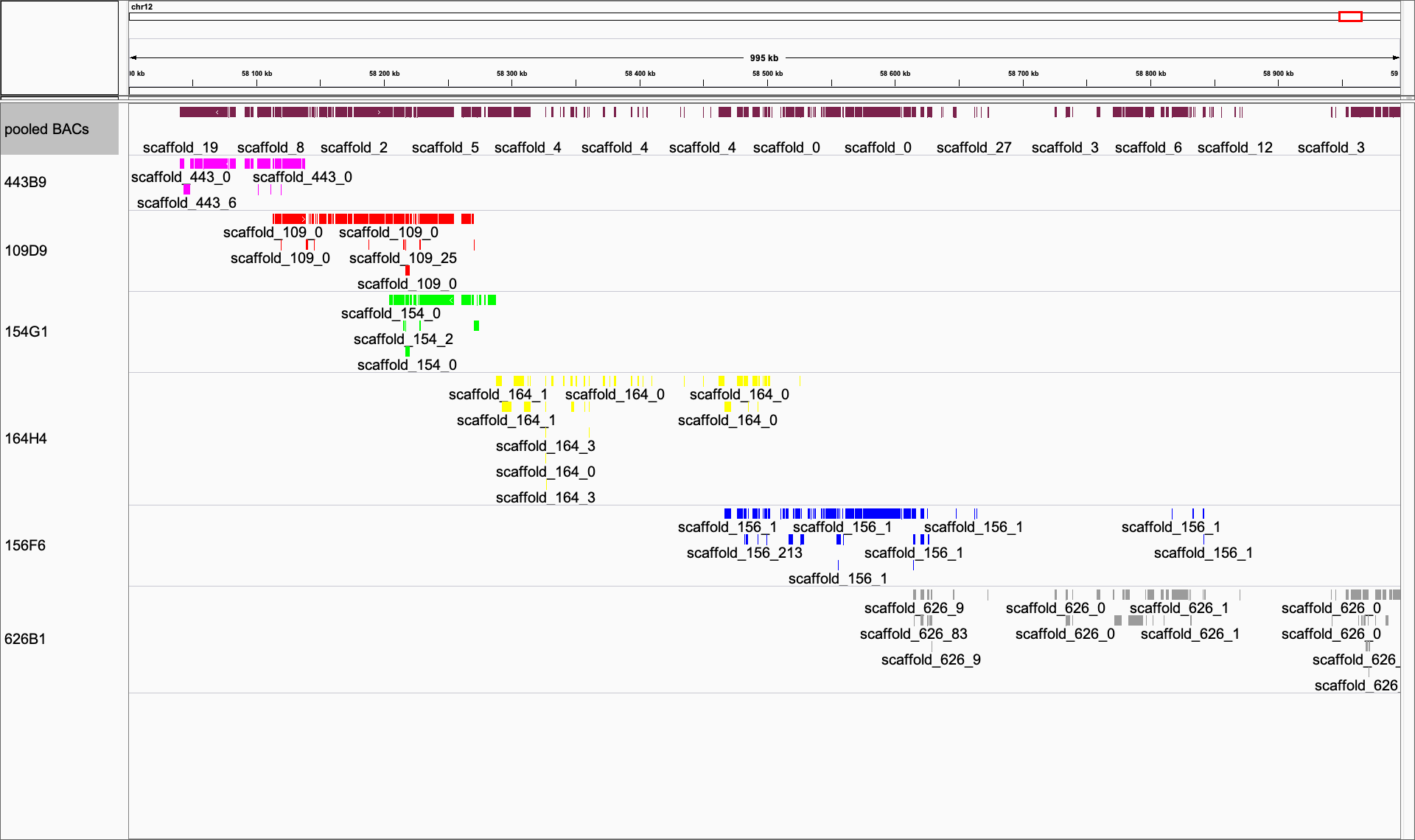
**

**Fig. S3. BAC reads of *Ry_sto_* locus aligned to the Phureja genome.** *De novo* scaffolds (longer than 1 Kb) of pooled and individual BAC reads were aligned into the reference genome Phureja using the BWA-MEM program. Only those fragments smaller than 1 Kb were aligned, which had higher than 90% similarity to Phureja. These fragments were converted into BED format and displayed in the IGV genome browser. The six BAC clones covers the *Ry_sto_* region between 58 and 59 Mb in the chromosome XII.
