# Supplementary material for: Mapping and DNA sequence characterisation of the Rysto locus conferring extreme virus resistance to potato cultivar ‘White Lady’
Source: PLoS One. 2020 Mar 31;15(3):e0224534. doi: 10.1371/journal.pone.0224534 (PMC7108733; doi:10.1371/journal.pone.0224534)
Supplement: S4 Fig — (DOCX) [file pone.0224534.s005.docx]

Chr1 GCCTCTCACTTTCAGGCTATGGATTAATAGGTGAGTTTCCTCGTGGCATTCAAAATTGTA 60

Chr2 GCCTCTCACTTTCAGGCTATGGATTAATAGGTGAGTTTCCTCGTGGCATTCGAAATTGTA 60

Chr3 GCCTCTCACTTTCAGGCTATGGATTAATAGGTGAGTTTCCTCGTGGCATTCGAAATTGTA 60

Chr4 GCCTCTCACTTTCAGGCTATGGATTAATAGGTGAGTTTCCTCGTGGCATTCAAAATTGTA 60

*************************************************** ********

Chr1 CAAGCTTGACAAGTTTAGATCTTTCAGGGAACAGCTTGTATGGAACTATCCCTTCTGATA 120

Chr2 CAAGCTTGACAGGTTTAGATCTTTCAGGGAACAGCTTGTATGGAACTATCCCTTCTGATA 120

Chr3 CAAGCTTGACAAGTTTAGATCTTTCAGGGAACAGCTTGTATGGAACTATCCCTTCTGATA 120

Chr4 CAAGCTTGACAAGTTTAGATCTTTCAGGGAACAGCTTGTATGGAACTATCCCTTCTGATA 120

*********** ************************************************

Chr1 TTTCAGCAATAGTTGAACATATTACAATACTTGATCTCTCAAATAACACGTTTTCGGGTG 180

Chr2 TTTCAGCAATAGTTGAACATGTTACAATACTTGATCTCTCAAATAACACGTTTTCGGGCT 180

Chr3 TTTCAGCAATAGTTGAATATGTTACTACACTTGATCTCTCAAATAACACGTTTTCGGCTG 180

Chr4 TTTCAGCAATAGTTGAACATATTACAATACTTGATCTCTCAAATAACACGTTTTCGGGTG 180

***************** ** **** * *****************************

Chr1 ATATACCACCTGATATAGCTAATTGTCAATACCTTAATGGTCTAAAGTTGGATAATAACT 240

Chr2 ATATACCACCTGATATAGCTAATTGTCAATACCTTAATGGTCTAAAGTTGGATAATAATT 240

Chr3 ATATACCACCTGATATAGCTAATTGTCAATACCTTAATGGTCTAAAGTTGGATAATAATT 240

Chr4 ATATACCACCTGATATAGCTAATTGTCAATACCTTAATGGTCTAAAGTTGGATAATAACT 240

********************************************************** *

Chr1 ATCTAGAAGGTGAAATTCCAAGCAGAATAGGCTATTTGCCTCGCCTTAAGATGTTCAGTG 300

Chr2 ATCTAGAAGGTGAAATTCCAACCAAAATAGGCTATTTGCCTCGCCTTAAGACGTTCAGTG 300

Chr3 ATCTAGAAGGTGAAATTCCAAGTAGAATAGGCTATTTGCCTCGCCTTAAGACGTTCAGTG 300

Chr4 ATCTAGAAGGTGAAATTCCAAGCAGAATAGGCTATTTGCCTCGCCTTAAGACGTTCAGTG 300

********************* * ************************** ********

Chr1 TAGCCAACAATTACTTGACTGGGCCAGTGCCATTGTTTTATAGCGAATATATCACAGCTG 360

Chr2 TAGCCAACAATTACTTGACTGGGGCAGTGCCATCGTCTTTTAGCGAATATATCACAGCTG 360

Chr3 TGGCCAATAATTACTTGACTGGGCCAGTGCCATCGTCTTTTAGCGAATATATCACAGCTG 360

Chr4 TGGCCAATAATTACTTGACTGGGCCAGTGCCATCGTCTTTTAGCGAATATATCACAGCTG 360

* ***** *************** ********* ** ** ********************

Chr1 GGAGTTTTGAAAACAATTCAGAGCTTTGTGGGAAGCCCTTGAAAGGATGTACTGAGGATT 420

Chr2 AGAGTTTTGAAAACAATTCAGAGCTTTGTGGGAAGCCCTTGAAAGCATGTG---AGGATT 417

Chr3 AGAGTTTTGAAAACAATTCAGAGCTTTGTGGGAAGCCCTTGAAAGGATGTACTGAGGATT 420

Chr4 AGAGTTTTGAAAACAATTCAGAGCTTTGTGGGAAGCCCTTGAAAGGATGTACTGAGGATT 420

******************************************** **** ******

Chr1 CTTGGATATCGAAACATGTAGATCGTGCTTCGTTCATCA 459

Chr2 CTTGGATATGGAAACATGTAGATCGTGCTTCGTTCATCA 456

Chr3 CTTGGATATGGAAACATGTAGATCGTGCTTCGTTCATCA 459

Chr4 CTTGGATATGGAAACATGTAGATCGTGCTTCGTTCATCA 459

********* *****************************

**Fig. S4.** **DNA sequence alignment of four homologous ‘White Lady’ genomic regions including Chr2 with 100% identity to the end sequences of BAC clones 154G1 and 164H4.** Overlapping nucleotides of the BAC clones are in red. The chromosomal regions Chr1-4 were PCR amplified using the primers BAC154-3 Fw and BAC164-5 R (Supplementary Table 1) and cloned into pGEM-T Easy (Promega, San Luis Obispo, CA, USA). Twenty-one clones were sequenced by the company BIOMI (Gödöllő, Hungary). NCBI Blast alignment assigned the sequences to four categories presumably corresponding to four allelic chromosomes in the tetraploid ‘White Lady’ genome: 7 clones were assorted to Chr1, 7 clones to Chr2, 5 clones to Chr3 and 2 clones to Chr4. The alignment was generated using the web tool Clustal Omega.
