# Supplementary material for: Mapping and DNA sequence characterisation of the Rysto locus conferring extreme virus resistance to potato cultivar ‘White Lady’
Source: PLoS One. 2020 Mar 31;15(3):e0224534. doi: 10.1371/journal.pone.0224534 (PMC7108733; doi:10.1371/journal.pone.0224534)
Supplement: S5 Fig — (DOCX) [file pone.0224534.s006.docx]

**BAC 443B9**

Scaffold 443-0 36141 GAAGAAGTGTTGGAGGGTCG 36160

||||||||||||||||||||

**1.109Fw** 20 GAAGAAGTGTTGGAGGGTCG 1

Scaffold 443-0 35981 ATACACCCAGCAGATTCGTCA 36001

|||||||||||||||||||||

**1.109R** 21 ATACACCCAGCAGATTCGTCA 41

**BAC 109D9**

Scaffold 109 29624 TGACGAATCTGCTGGGTGTAT 29644

|||||||||||||||||||||

**1.109R** 41 TGACGAATCTGCTGGGTGTAT 21

Scaffold 109 29465 CGACCCTCCAACACTTCTTC 29484

||||||||||||||||||||

**1.109Fw**  1 CGACCCTCCAACACTTCTTC 20

Scaffold 109-0 15472 ATGCGGGAGAACACGATAC 15490

|||||||||||||||||||

**ST1Fw** 1 ATGCGGGAGAACACGATAC 19

Scaffold 109-0 15746 CATCCCACATTGCCTCACA 15764

|||||||||||||||||||

**ST1R**  38 CATCCCACATTGCCTCACA 20

**BAC 154G1**

Scaffold 154-0 34423 ATGCGGGAGAACACGATAC 34441

|||||||||||||||||||

**ST1Fw**  1 ATGCGGGAGAACACGATAC 19

Scaffold 154-1 34697 CATCCCACATTGCCTCACA 34715

|||||||||||||||||||

**ST1R**  38 CATCCCACATTGCCTCACA 20

**BAC 164H4**

Scaffold 164-0 31797 TAAGGTAATGAACACATGCA 31816

|||||||||| || ||||||

**1.110Fw** 20 TAAGGTAATGCACCCATGCA 1

Scaffold 164-0 31063 CATTTTATGTTGACCCATTTTC 31084

||||| |||||||||||| |||

**1.110R** 21 CATTTGATGTTGACCCATGTTC 42

**BAC 156F6**

Scaffold 156-1 98232 ACACACGTCCTTCTTGCGCGT 98253

|||||||||||||||||||||

**DisResFw**  22 ACACACGTCCTTCTTGCGCGT 1

Scaffold 156-1 97194 TAGCTCAACGCGAGGACACCAT 97215

||||||||||||||||||||||

**DisResR** 21 TAGCTCAACGCGAGGACACCAT 42

Scaffold 156-1 17917 ATCGTCTGCAAGCTCTTGGG 17936

||||||||||||||||||||

**1.156Fw** 1 ATCGTCTGCAAGCTCTTGGG 20

Scaffold 156-1 18453 GTTCATATTGCATGCGGGGC 18472

||||||||||||||||||||

**1.156R** 40 GTTCATATTGCATGCGGGGC 21

**BAC 626B1**

Scaffold 626-0 29991 TTCACAACTTGACCCTCGGATT 30012

||||||||||||||||||||||

**1.365Fw** 1 TTCACAACTTGACCCTCGGATT 22

Scaffold 626-0 30448 AGTAGCCTATGCTCAGCAAAAT 30469

||| ||||||||||||||||||

**1.365R** 41 AGTTGCCTATGCTCAGCAAAAT 20

**Fig. S5. DNA sequence identity of the *Ry_sto_* region specific primers with the corresponding BAC clone sequences.** The alignment was generated using NCBI BLAST.
