# Supplementary material for: Mapping and DNA sequence characterisation of the Rysto locus conferring extreme virus resistance to potato cultivar ‘White Lady’
Source: PLoS One. 2020 Mar 31;15(3):e0224534. doi: 10.1371/journal.pone.0224534 (PMC7108733; doi:10.1371/journal.pone.0224534)
Supplement: S6 Fig — (DOCX) [file pone.0224534.s007.docx]

DisRes 1 MYDSSLFGLLQMLYETLKNEAKFLSNVSNQIQDIHAEINRIQCFLQDADAKKPEYETVRN
RPP8 1 MYDSSLFGLLQMLYETLKNEAKFLSNVSNQIQDIRAEINRIQCFLQDADAKKPEYETVRN

DisRes 61 WIADIREVAYDVENILEKYMHKVALRKDRSLWKENINLHNIGLETKDVMSRIDNIKRCMK
RPP8 61 WIADIREVAYDVENILEKYMHKVALRKDRSLWKENINLHNIGLETKDVMSRIDNIKRCMK

DisRes 121 TYVDTGIRAICQGDISSERSQWLTRSYSHLVDEDFVGLVEEVNKLVDELINDENDEFYVV
RPP8 121 TYVDTGIRAICQGDTSSERSQWLTRSYSHLVDEDFVGLVEEVNKLVDELINEENDEFYVV

DisRes 181 FAICGMGGLGKTTLARKAYRHGDVQSHFQAFAWASISRQWQARDVLLSILMKLEPENRTR
RPP8 181 FAICGMGGLGKTTLARKAYRHGDVQSHFQAFAWASISRQWQARDVLLSILMKLEPENRTR

DisRes 241 INMMMDDELVKALYNVQQRKRCLIVLDDIWSTNFWNSVKHAFPKGNGSRSKILLTTRKKD
RPP8 241 INMMMDDELVKALYNVQQRKKCLIVLDDIWSTDFWNSVKHAFPKGKGSRSKILLTTRKKD

DisRes 301 VCTHIDPTCFLFEPRCLDAEESWKLLHKKAFPRVNTPDLKIDLELERLGKEMVSKCGGLP
RPP8 301 VCTHIDPTCFLFEPRCLDAEESWKLLHKKAFPGVNTPDL-IDLELERPGKEMVSKCGGLP

DisRes 361 LAIIVLAGLLARRPKIDEWRRTCQNLNLHMSGESFEQDGGIHGVLALSYYDLPYQLKPCF
RPP8 360 LAIIVLAGLLARRPKIDEWRRTCQNLNLHMSGESFEQDGGIHGVLALSYYDLPYQLKPCF

DisRes 421 LYLGNFPEDQKISARRLYQLWAAEGIIPLEGNRGEETTMMERGERYLHELAQRYMVQVQL
RPP8 420 LYLGNFPEDQKISARRLYQLWAAEGIISLEGNRGEETAMMERGERYLHELAQRYMVQVQL

DisRes 481 EETTGRIKSCRFHDLMRDTCLSKAKEENFLKTVSPQHLHQSMHCSTSATATLTRTVRRLS
RPP8 480 EETTGRIKSCRFHDLMRDTCLSKAKEENFLKTVSPQHLHQSMHCSTSATATSTRTVRRVS

DisRes 541 ITVDNEVQNYFSTDDKSFQHVRSALFFPIQTGREGTEYPLPLFQGLCNNFAMLRVLHLEK
RPP8 540 ITVDNEVQNYFSTDDKSFQHVRSALFFPIQTGREGTEYPLPLFQGLCNNFSMLRVLHLEK

DisRes 601 FTFVEILPKAIGNLVYLRYLSLRHSHFQKLSSSVGNLKYLQTLDLRVNFFSYLTLPNTIQ
RPP8 600 FTFVEILPKAIGNLVYLRYLSLRHSHFQKLSSSVGNLKYLQTLDLRVNFFSYLTLPNTIQ

DisRes 661 KLQNLRNLYLPPSHQHTYKLDLSPLSHLEILKNFDTQVSPFRDLFKLTKLQKLSAVLSLE
RPP8 660 KLRNLRNLYLPPSHQHTYKLDLSPLSHLEILKNFDTQVSPFQDLFKLTKLQKLSAVLSLE

DisRes 721 SYEMEEMIKHLNLRSGRLREASFRIYYRFHSEKEVNILKLLLGCPHLRKLDLIGHITKLP
RPP8 720 SYEMEEMIKHLNLRSGRLRETSFRIYYRFHSEKEVNILKLLLGCHHLRKLDLIGHITQLP

DisRes 781 EHHSFSQSLTKLTLRKSGLEEDPMVILQKLPKLFTLSLRGNAFIGKEMCCSPQGFPLLKT
RPP8 780 EHHSFSQSLTKLTLRKSGLEEDPMVILQKLPKLFSLSLRGNAFIGKEMCCSPQGFPLLKT

DisRes 841 LKLQGLPNLESWRVETGALPNLVHLEIDECKKLEMVPEGLIYLTKIQEVMIINMPDNFQN
RPP8 840 LKLQGLLNLESWRVEKGALPNLVHLEIDECKKLEMVPEGLIYLTKIQEVMIINMPENFQN

DisRes 901 RLQEVQREEYYKVQFRKNFDTKKISKIKFNMRISGSGPVFGGLLQTIYPSTVPKEWKML
RPP8 900 RLQEVQREEFYKVQFRKNFDTQKISKIKFKMRIS--GPMFGGLLQTIYPSTVPKEWKML

**Fig. S6. Amino acid alignment of the putative disease resistance protein DisRes with the disease resistance RPP8-like protein 2** (XP_015162129.1) derived from a genomic sequence annotated using gene prediction method: Gnomon. The alignment was generated using the web tool Clustal Omega.
