# Supplementary material for: Mapping and DNA sequence characterisation of the Rysto locus conferring extreme virus resistance to potato cultivar ‘White Lady’
Source: PLoS One. 2020 Mar 31;15(3):e0224534. doi: 10.1371/journal.pone.0224534 (PMC7108733; doi:10.1371/journal.pone.0224534)
Supplement: S7 Fig — (DOCX) [file pone.0224534.s008.docx]

Phloem1 1 MSHTFSSEIHKYDAFLSFRGEDTRRTFVSHLYNALIQGRIDVFKDDERLETGKSISDELP
Phloem2 1 MSHTSSSKIQKYDAFLSFRGVDIRKTFVSHLYNALIQGGINVFKDDERLETGKSISDELP
Phloem4 1 MSHTSSSKIHKYDVFLSFKGNDTRRTFVSHLYNALIERRIDVFKDDDRLETGKSIYDELP
PhloemA5 1 MSHTFSSEIHKYDAFLSFRGEDTRKTFVSHLYNALIQGRIDVFKDDERLETGNSISDELP


Phloem1 61 KAIEESKFAIVIFSESYASSKWCLEELAHIIKCRKELKQIVIPIFYNVDPLDVRHQTQTF
Phloem2 61 KAIEESKFAIVIFSKSYASSKWCLDELAHIIKCRKELDQIVIPIFYNVDPSDVRHQTQTF
Phloem4 61 KAIEESKFAIVIFSKRYASSKWCLDELAHIIKCRKELDQIVIPIFYNVNPSDVSHQNPPF
PhloemA5 61 KAIEESKFAIVIFSESYASSKWCLDELAHIIKCRKELKQIVIPIFYNVDPSDVRHQTQTF


Phloem1 121 AESFSQHEEKYKDDIEKIQRWRDAFAESGSISGYHLQNYKDEADCIKKVVERLKSVLHIE
Phloem2 121 AESFSKHEEKYKDDMEKIQSWRDAFAESGKIVGHHLQNYKDEADCIKKVVDELMSSLHIK
Phloem4 121 AESFSQHEEKYKDDIEKIQGWRDAFAESGKISGYHLQNYKDEADCIKKVVDKLMSLLHIE
PhloemA5 121 AESFSQHEEKYKDDMEKIQRWRDAFAESGKISGYHLQNYKDEADCIKKVVERLMSVLHIE


Phloem1 181 SDDDDVRAFIRGMDNNSPILIDGGKMSFSLDKKR-KKCFMLAARELDIEWSHEPKYWKWL
Phloem2 181 SDDDDD-AGDRGMDAKSPILIDEGKMSFSRDKKTGKKCFMIAARGLDIEWSNTPDYWEWL
Phloem4 181 SDDHYNDDVLKG--PKSPILIDGGKMSFWLDKKTGKKCFMVAARGLEIEWGGTLEYWEWL
PhloemA5 181 SDDDGVRAFIRGMDNNSPILIDGGKMSFSLDKKR-KKCFMIAARELDIEWSHKPKYWKWL


Phloem1 240 SHSD-SRFTEVAKLKKVYWLEIQGKIDSRRLSKRTKYVAYLVFKLENQFHGLETVNAVVR
Phloem2 240 PHSD------SSKLKWVCWLDIRGKIETRRLSKRTKYVAYLVFKLEDKFHGLETVNAVVR
Phloem4 239 SHSGLKRFAEVAKLKRVCWLDIRGKIETRRLSKRTKYVVYLVFKLENKWHGLETANAVVR
PhloemA5 240 SHSD-SRFAEVAKLKKVYWLEIQGKIDSRRLSKRTKYVAYLVFKLENQFYGLETVNAVVR


Phloem1 299 FVDSMSKKDAEKRASVVHFAGRGPRETLPFKRADGWMELKMGDFFNDAGEDGYVDAQLME
Phloem2 294 FVDSMSVKEAEQRASVVHFAGRGPRETLPFKRGDGWMELKMGEFFNDAGEDGDVDARLME
Phloem4 299 FVDSVSDIDAEQRASVVHFAGQGPKETLPFRRGDGWMELKMGDFFNDAGEDGDVDARLME
PhloemA5 299 FVDSMSKKDAEKRASVVHFAGRGPRETLPFERADGWMELKMGDFFNDAGEDGYVDARLME


Phloem1 359 TKKLDEKSGLIVQGVEFRPE
Phloem2 354 TKHLEAKGGLIVQGMEFRPE
Phloem4 359 TKHLGEKSGLIVQGVEFRPE
PhloemA5 359 TKKLDEKKGLIVQGVEFRPE

**Fig. S7. Amino acid alignment of phloem proteins.** Phloem proteins 1, 2 and 4 were identified in this study, while Phloem A5 (XP_015162041.1) is predicted from genomic sequence and supported by EST evidence. The alignment was generated using the web tool Clustal Omega.
