# Supplementary material for: Mapping and DNA sequence characterisation of the Rysto locus conferring extreme virus resistance to potato cultivar ‘White Lady’
Source: PLoS One. 2020 Mar 31;15(3):e0224534. doi: 10.1371/journal.pone.0224534 (PMC7108733; doi:10.1371/journal.pone.0224534)
Supplement: S8 Fig — (DOCX) [file pone.0224534.s009.docx]

N.t.TMV 1 MYLVKQQHNDQLSSKMSCVSSSMSSSSKVWKYDVFLSFRGEDTRKNFVSHLYNALEQRGI
TMV1 1 ------------------------------------------------------------
TMV2 1 M--------------------SHASSSKVCKYDIFLSFRGEDTRRNFVSHLFNALEQRGI
TMV3 1 M--------------------AHASSSKICKYDVFLSFRGEDTRRNFVSHLYNSLELRGI


N.t.TMV 61 HAFKDDERLETGKSISVELLKAIEESRFAVVIFSKRYASSKWCLEELAHIIKCRNELDQI
TMV1 1 ------------------------------------------------------------
TMV2 41 RTFKDDERLETGKSISTELLKAIEEARFAVIIFSKSYASSRWCLEELADIIKCKKELEQI
TMV3 41 RTFKDDERLEMGKSISDELLKAIQESKFAIVIFSKSYASSRWCLEELAHIIKCKKELEQI


N.t.TMV 121 VLPIFYDVSPSVVRHQNPPFTKSFSKHEETYKDDKEKVQRWRDAFAEAGKLSGHDLKNYK
TMV1 1 ------------------------------------------------------------
TMV2 101 VIPVFYDVSPSDVRHQNPPFAVSFSQHEEKCKDDMEKVQRWRGAFAEAGKISGYHLLNFK
TMV3 101 VIPVFYDVSPSDVRHQHPPFADSFLQHEEKCKDDMEKVQRWRGAFAEAGKISGYHLQNFK


N.t.TMV 181 DEVECINKVVDYILPKSLQVIPLSSGSLVGMEPQIGKIISLLDTESNDVRSIGLWGMSGI
TMV1 1 ------------------------------------------------------------
TMV2 161 DEAECVKKLVDDIFPKSLQIISPFPVNLVGMKSQVEKVTSLLDMESNDVRSIGIWGMGGI
TMV3 161 DDAECIKKLVDDIFPKSLQSISPFPESLEGMKSQVEKVISLLDMESNDVRSIGIWGMGGI


N.t.TMV 241 GKTEIASVIYERYRHQFEADCFLGDVGEMYLKKGLTWLQQALIHKLLGKNIPITSEREGA
TMV1 1 ------------------------------------------------------------
TMV2 221 GKTEIANILHQRYRHRFEADCFLGDVGKLHQKNGLTWLQQVVICKLLGEKLTLTSEHEGM
TMV3 221 GKTEIANVLHQTYRHQFEADCFLGDVGKFHQKNGLTWLQQVVICKLLGEKLTLTSEHEGM


N.t.TMV 301 IIIKNGLRWKKVLVILDDVSHLSQLELIVGGTEWFGRGSRILITTRDKHIIIAHVKEDKV
TMV1 1 ------------------------------------------------------------
TMV2 281 NILKNMLRWKKVLFIIDDVNHQEQLEFLVGEPEWFGRGSRIILTARDKHLLISHVG-DNV
TMV3 281 NILKNMLRWKKVLFTIDDVNHQEQLEFLVGEPEWFGRGSRIILTARDKHLLISHVG-DNV


N.t.TMV 361 YEVQLLSENDALELFYVHAFNRNSPERDFEELSREVVKYADGLPLALKVLGPSFCGRNKE
TMV1 1 ------------------------------------------------------------
TMV2 340 YEVQLLSEDEALELFSRHAFREKSPKEDFLELSSEVVEHAGGLPLALKVLGSSFYGRDKK
TMV3 340 YEVQLLSENEALELFSRHAFRERSPKEDFLELSRQVVNHAGGLPLALKDLGSSFYRRDKK


N.t.TMV 421 QWRDIIDRLKKIPNDDILGKLKIGLDGLNRDEMRIFLDIASLYN-----YKSMDHVALIL
TMV1 1 ------------------------------------------------------------
TMV2 400 HWRHIIDRLKRIPHKDILGKLRLSFDCLDKDEKELFLDIVFLYIARLSSYDFDICVEQVQ
TMV3 400 HWRPIIDRLKIIPHKDILGKLRLSFDGLDKDEKELFLDIVFLDIACLTRYDFHLCAEQIL


N.t.TMV 476 KSCGIHQSIGISRLIEKSLLSFSRYDYTFRMHSLIRKMGENMLREEYANSRIWLHEEVND
TMV1 1 -------------------------------------MGENVIREEYANSKIWLPEEVCD
TMV2 460 RY--VSRGFLIDYLIEKSLLSNDLNN-SIVMHNMIREMGENVIREEYANSRIWLPEEVCD
TMV3 460 RY--LSHGFLIDYLIEKSLLSINLYN-SIVMHNMIREMGENVIRKEYANSRIWLLKEVCD


N.t.TMV 536 LFAGKLKTKKVESLWIPKGFDFEDDRVNHSKVFKRMKSLQVLILGETVWSDFLFLVMAET
TMV1 24 LFKGKLIKEKVESLCIPQGCYFEDDLDNYSNIFKRMQSLKILIVGDGTFS----------
TMV2 517 LFKGKLITEKVESLCIPKEYYFDDDFVDYGNIFKRMQSLQILIVGNGTFS----------
TMV3 517 LFKGKLITEKVESLCIPKEYYFDDDFVDYSNIFKRMQSLQILIVGNGTFS----------


N.t.TMV 596 ICSRSIITCLPSSLRWIEWPNYPSRLLPERFEPSHLVGLCLKGSRLVELWPISKRLSNLK
TMV1 74 --SNCAVTYLPSSLRLIDWLGYPSISLPESFEPSQLVVLCLHKSWLVELLPISKKLSNLK
TMV2 567 --SNCAITYLPSSLRFIDWSGYPSISLPESFEPSQLAMLCLCESRLVELWAISKKLSNLK
TMV3 567 --SNCAITYLPSSLRFIDWKGYPSISLPESFEPSQLAMLCLRKSRLVELLPISKKLSNLK


N.t.TMV 656 HLDLSKSLGLRKTPSFGDMPNLERLILEGCKNLEEVHSSLGHCRMLTSLNLRGCSKLKKL
TMV1 132 HLDLMDSCELRKTPTFGDMPNLETLILHGCVNLEEVHPSLGHCRLLTYLSLEGCRKLKKL
TMV2 625 HLDLMGSCELRKTPNFGDMPNLEKLYLSGCVNLEEVHPSLGHCRMLTSLSLSDCHKLQKL
TMV3 625 HLDLMDRCELRKTPNFGDMPNLEKLYLRGGVNLEEQLEV--------------------V

N.t.TMV 716 PKFVSMESLETLNLRECTSLRKFPKICGNMQRLSELYVESPWIRSLPLMSLSGLSKLHLY
TMV1 192 PKFVCVESLETLNLLECTSLQEFPEICGDMPCLSVLSVKSPWIRSLP-PSFSSLRNLELT
TMV2 685 PKFVCMDSLEDLDLSECTRLEEFPEICGDMHGLSILYLGSPWIRSLP-PSFSSLRNLQLT
TMV3 665 DKY---------------------------------------------GSLSNLRRLRLT


N.t.TMV 776 YCEDLESIPDTIIQNLRYLDILGCNKLATLPNSLSESEQLEQLSIHRCSRLVELPISLRV
TMV1 251 ECEVLESIPDA-IQNLRYLSISGCNKLATLPNSLFESQQLEYLLICQCSGLVKLPISLGV
TMV2 744 DCEVLESIPDA-IQNLRYLSISGCNKLATLPNNLFESQQLEYLLIWQCSGLVKLPISLGV
TMV3 680 DCEILECIPDT-IQNLSDLSISGCNKLATLPNSLFESQQLNYLKIHRCSGLVKLPISLGV


N.t.TMV 836 QRKLVRLALDRCENLKKLPKSIQMESLGYLGIYNCPRLDTFPEINGDMRCLKHLTVNSTE
TMV1 310 QKKMCWLEIDGCENLKKLPSSIQMKSLKKLKIANSPKLDTFPEIDGDMHYLTSLILNSTG
TMV2 803 QKILRWLDIDGCENLKKLPSSIQMKSLQKLEIANSPKLDTFPEINGDMHCLKELTLNSTG
TMV3 739 QKNLCGLEIDGCENLKKLPSSIQMKSLEKLRISNSPKLDTFPEIDGDMHYLTQLTLNSTG


N.t.TMV 896 IRELPSSIGNLSGLNTLNLEGCEDLASLPNSLCNLTNLQSLILYGCKKLENLPENIGDLQ
TMV1 370 IREVPSSTEHLRGLEYLYLSGCEDLLSLPDSLCNLMKLQSLYLDGCKKLEKLPENIGDLQ
TMV2 863 IREVPSSIGNLSGLTELNLTGCEDLLSLPDSLCNLMKLQSLYLDGCKKLEKLPENIGDLQ
TMV3 799 IREVPSSIGNLSGLTLLKLSGCEDLVSLPDSICNLMNLQRLFLGRCRKLEKLPENMGDLQ


N.t.TMV 956 QLRLLDARGTAISQPPPSIIKLGKLWSFRFSHVVQLQHSSSFVLHQLSALSSLTHLYLNN
TMV1 430 DLRILDASETAISQPPPSITKLGKLWTLRFSHEQQFQHSSSFVLNQVSGLSSLTSLDLNN
TMV2 923 DLHILDASDTAISQPPSSITKLGKLWKLRFSHEKQLQYSSSFVLNQ--------------
TMV3 859 ELCMLDASKTAIYQLPPSITKLGKLWTLRFSHEKQLRHSSSFVLHQISGLSSLQELHLNN


N.t.TMV 1016 LNMLDGLPEDLGSLHFLEYLNVSGSNISCLPNSINELLCLECLNVQFCKGLTELPGELPP
TMV1 490 HNIMSGLPEDLGSLHSLENLNCD-------------------------------------
TMV2 ------------------------------------------------------------
TMV3 919 LNILGGLPEELGSLRKLD------------------------------------------


N.t.TMV 1076 NLRVLCADYHLALKSIRDLVFKCVNLEEISISWCGHEITECRTVTSNQVNVFKFLHHFLR
TMV1 513 ------------------------------------------------------------
TMV2 ------------------------------------------------------------
TMV3 ------------------------------------------------------------


N.t.TMV 1136 RSIQNDFFRQRWIRFSISFPQGKIPEFFTY-QFINQNRISVNLNPSWYTDKFMGFSVCYQ
TMV1 513 -------FHQR-AYFIISFPGVRIPKLFDYDRFINQNEISIELNPSWYTDKFMGFWISYG
TMV2 ------------------------------------------------------------
TMV3 ------------------------------------------------------------


N.t.TMV 1195 VHGGEKDSKVTPTLVCRLSGLETLLGSEDPLCLYE-------SPNDNPAPGMLFIYIPFQ
TMV1 565 P--TRLDAGLEATLVCKS----------DPERKYSLEYNYFPEYSRFEYPFTCCAYIPFE
TMV2 ------------------------------------------------------------
TMV3 ------------------------------------------------------------


N.t.TMV 1248 IFRDHFKPLGTKAKNPNDYCLFEVS-VMSGKEGCWGIRLEYENKVRRWRRKQRVTQSPKL
TMV1 613 TLWNAS--DNKEGRNPNDYYMLEVPEVYREEELCWGIRLEYEKEAM--------------
TMV2 ------------------------------------------------------------
TMV3 ------------------------------------------------------------


N.t.TMV 1307 HPVPQKDNAVTTEIGCSMAFKQQEHSSCTSSSQAFVENNIATERGLHLYYENKDLEVDQA
TMV1 657 ------------------------------------------------------------
TMV2 ------------------------------------------------------------
TMV3 ------------------------------------------------------------


N.t.TMV 1367 ATVVQKEHESQNVELIGVCDKPLRKMDDAPRKRKKKRKMAGENENQRACMTLIGS
TMV1 657 --------------------------SDAK-------------------------
TMV2 -------------------------------------------------------
TMV3 -------------------------------------------------------

**Fig. S8. Amino acid alignment of TMV resistance proteins.** TMV1-3 were identified in this study, while N.t.TMV (XP_016461601.1) is a predicted protein of *Nicotiana tabacum* derived from a genomic sequence annotated using gene prediction method: Gnomon. The alignment was generated using the web tool Clustal Omega.
