# Supplementary material for: Mapping and DNA sequence characterisation of the Rysto locus conferring extreme virus resistance to potato cultivar ‘White Lady’
Source: PLoS One. 2020 Mar 31;15(3):e0224534. doi: 10.1371/journal.pone.0224534 (PMC7108733; doi:10.1371/journal.pone.0224534)
Supplement: S9 Fig — (DOCX) [file pone.0224534.s010.docx]

TMV2 1 MSHASSS-----------KVCKYDIFLSFRGEDTRRNFVSHLFNALEQRGIRTFKDDERL
Y-1 1 MASSSSSSESNSQYSCPQRKYKYDVFLSFR-------------------------DDKRL

TMV2 50 ETGKSISTELLKAIEEARFAVIIFSKSYASSRWCLEELADIIKCKKELEQIVIPVFYDVS
Y-1 36 ENGDSLSKELVKAIKESQVAVIIFSKNYATSRWCLNEVVKIMECKEENGQLVIPVFYDVD

TMV2 110 PSDVRHQNPPFAVSFSQHEEKCKDDM---EKVQRWRGAFAEAGKISGYHLLNFKDEAECV
Y-1 96 PSDVRKQTKSFAEAFAEHESRYKDDVEGMQKVQRWRTALSEAADLKGYDIR-ERIESECI

TMV2 167 KKLVDDIFPKSLQIISPFPVNLVGMKSQVEKVTSLLDMESNDVRSIGIWGMGGIGKTEIA
Y-1 155 GELVNEISPKLCETSLSYLTDVVGIDAHLKKVNSLLEMKIDDVRIVWIWGMGGVGKTTIA

TMV2 227 NILHQRYRHRFEADCFLGDVGKLHQKNGLTWLQQVVICKLLGEKLTLTS-EHEGMNILKN
Y-1 215 RAIFDILSSKFDGACFLPDNK--ENKYEIHSLQSILLSKLVGEKENCVHDKEDGRHLMAR

TMV2 286 MLRWKKVLFIIDDVNHQEQLEFLVGEPEWFGRGSRIILTARDKHLLISHVGDNVYEVQLL
Y-1 273 RLRLKKVLVVLDNIDHEDQLKYLAGDLGWFGNGTRIIATTRDKHFIR--KNDAVYPVTTL

TMV2 346 SEDEALELFSRHAFREKSPKEDFLELSSEVVEHAGGLPLALKVLGSSFYGRDKKHWRHII
Y-1 331 LEHDAVQLFNQYAFKNEVPDKCFEEITLEVVSHAEGLPLALKVWGSSLHKKDIHVWRSAV

TMV2 406 DRLKRIPHKDILGKLRLSFDCLDKDEKELFLDIVFLYIARLSSYDFDICVEQVQRYVSRG
Y-1 391 DRIKRNPSSKVVENLKVSYDGLEREDQEIFLDIACFLRGRKQT-EIKQILESCDFGADDG

TMV2 466 FLIDYLIEKSLLSNDLNNSIVMHNMIREMGENVIREE---YANSRIWLPEEVCDLFKGKL
Y-1 450 LR--VLIDKSLVFISEYDTIQMHDLIQEMGKYIVTMQKDRGEVTRLWLTQDFEKFSNAKI

TMV2 523 I-TEKVESLCIPKEYYFDDDFVDYGNIFKRMQSLQILIVGNGTFSSNCAITYLPSSLRFI
Y-1 508 QGTKAIEAIWIPEIQ----DLSFRKKAMKDVEKLRILYINGFHTPDGSNDQYLPSNLRWF

TMV2 582 DWSGYPSISLPESFEPSQLAMLCLCESRLVELWAISKKLSNLKHLDLMGSCELRKTPNFG
Y-1 564 DCCKYPWESLPAKFDPDMLVHLDLQQSSLFHLWTGTKKFPFLRRLDLSSCANLMRTPDFT

TMV2 642 DMPNLEKLYLSGCVNLEEVHPSLGHCRMLTSLSLSDCHKLQKLPKFVCMDSLEDLDLSEC
Y-1 624 DMPNLEYLGLEECSNLKEVHHSLRCSKKLIKLNLRDCKNLESF-SYVCWESLECLHLQGC

TMV2 702 TRLEEFPEICGDMHGLSILYLGSPWIRSLPPSFSSLRNLQLTDCEVLESIPDAIQNLRYL
Y-1 683 SNLEKFPRIRGKLKPEIEIQVQRSGIRKLPSAI-----------------IQHQSSLTEL

TMV2 762 SISGCNKLATLPNNLFESQQLEYLLIWQCSGLVKLPISLGVQKILRWLDIDGCENLKKLP
Y-1 726 DLSGMKNLATLSCSIGELKSLVMLKVSYCSKLKSLPEEIGDLENLEILK-AGYTLISQPP

TMV2 822 SSIQ-MKSL-------QKLEIANSPKL-DTFPEINGDMHCLKELTLNSTGIRE--VPSSI
Y-1 785 SSIVRLNRLKFLTFAKQKSEVGLEDEVHFVFPPVNQGLCSLKTLNLSYCNLKDEGLPQDI

TMV2 871 GNLSGLTELNLTGCEDLLSLPDSLCNLMKLQSLYLDGCKKLEKLPENIGDLQDLHIL---
Y-1 845 GSLSSLEVLNLRG-NNFEHLPQSLTRLSSLQSLDLLDCKSLTQLPEFPRQLDTIYADWNN

TMV2 928 ------------------------------------------------------------
Y-1 904 DSICNSLFQNISSFQHDICASDSLSLRVFTNEWKNIPRWFHHQGKDKSVSVKLPENWYVC

TMV2 928 -----------------------------------------------------------D
Y-1 964 DNFLGFAVCYSGCLIETTAQFLCDEGMPCITQKLALPKHSEEFPESAIHFFLVPSAGLLD

TMV2 929 ASDTAISQPPS-------SI----------------TKLGKLWKLRFSHEKQLQYSSSFV
Y-1 1024 TSKANGK-TPNDYRHIMLSFSEELKEFGLRLLYRDESKLKALFKMTENNDEPTEYCVVKR

TMV2 966 LNQ----------------

Y-1 1083 RGQYDEARCSSSKKQRSQL

**Fig. S9. Amino acid alignment of proteins encoded by *TMV2* isolated from the *Ry_sto_*-bearing potato cultivar ‘White Lady’ and Y-1** (CAC82812.1) encoded by a gene co-segregating with *Ry_adg_*, an ER gene originating from *S. andigena* (Vidal et al. 2002). The alignment was generated using the web tool Clustal Omega.
