# Supplementary material for: Mapping and DNA sequence characterisation of the Rysto locus conferring extreme virus resistance to potato cultivar ‘White Lady’
Source: PLoS One. 2020 Mar 31;15(3):e0224534. doi: 10.1371/journal.pone.0224534 (PMC7108733; doi:10.1371/journal.pone.0224534)
Supplement: S10 Fig — (DOCX) [file pone.0224534.s011.docx]

TMV2 GGTGCTAAGAAGACTTCATATCAGATACGCGTCTTTAGCAATATTCTTCAACAATGTCTC 60

Rysto GGTGCTAAGAAGACTTCATATCAGATACGCGTCTTTAGCAATATTCTTCAACAATGTCTC 50

************************************************************

TMV2 ATGCTTCTTCTTCCAAAGTTTGCAAGTACGATATCTTTTTGAGTTTTAGAGGTGAAGATA 120

Rysto ATGCTTCTTCTTCCAAAGTTTGCAAGTACGATATCTTTTTGAGTTTTAGAGGTGAAGATA 110

************************************************************

TMV2 CGCGTAGAAACTTCGTGAGTCATCTTTTTAATGCTTTAGAACAGAGAGGAATCCGCACTT 180

Rysto CACGTAGAAACTTCGTGAGTCATCTTTATAATGCTTTAGAACAGAGAGGACTCCATGCTT 170

* ************************* ********************** *** ***

TMV2 TTAAAGATGATGAGCGACTAGAAACGGGAAAATCAATTTCTACTGAACTTTTAAAAGCCA 240

Rysto TCAAAGACGATGAGCGGTTGGAAGCAGGAAAATCAATTTCTGCTGAACTTTTAAAAGCCA 230

* ***** ******** * *** * *************** ******************

TMV2 TAGAAGAGGCCAGATTCGCTGTCATAATATTCTCAAAAAGCTATGCATCGTCAAGATGGT 300

Rysto TAGAAGAGGCCAGATTCGCTGTCGTAATATTTTCAAAAAGCTATGCATCGTCAAGATGGT 290

*********************** ******* ****************************

TMV2 GTTTAGAGGAGCTTGCAGACATCATAAAGTGTAAAAAGGAATTGGAGCAGATTGTGATTC 360

Rysto GTTTAGAGGAGCTTGCACACATCATAAAGTGTAAAAAGGAATTGGAGCAGATTGTGATTC 350

***************** ******************************************

TMV2 CAGTCTTTTATGATGTGAGTCCATCAGATGTACGCCATCAAAATCCCCCTTTCGCTGTTT 420

Rysto CAGTCTTCTATGATGTGAGTCCATCAGATGTACGCCATCAAAATCCCCCTTTCGCTGTTT 410

******* ****************************************************

TMV2 CATTTTCCCAACATGAGGAAAAATGCAAAGATGATATGGAGAAGGTTCAAAGATGGAGGG 480

Rysto CATTTTCCCAACATGAGGAAAAATGCAAAGATGATATGGAGAAGGTTCAAAGATGGAGGG 470

************************************************************

TMV2 GCGCATTTGCGGAGGCAGGGAAAATATCAGGCTATCATTTACTAAATTTCAAGTAAGCCT 540

Rysto GCGCATTTGCGGAGGCAGGGAAAATATCAGGCTATCATTTACTAAATTTCAAGTAAGCCT 530

************************************************************

TMV2 TCTTCTTTTTCTTTTTTTTCGGGTTTTTCTTTATCAAGAGGAAAAAGGACCAAACTTTTA 600

Rysto TCTTTTTT--CTTTTTTTTCGGGTTTCTCTTTATCAAGAGGAAAAAGGACCAAACTTTTA 588

**** *** **************** *********************************

TMV2 CCGAAAAAGGAACAAAGATCCTAGGAAAACAGAGACGTATATGATTGTTTGATGATGTAA 660

Rysto GCCAAAAAGGAACAAAGATCCTAGGAAAACAGAAACGTATATGATTGTTTGATGATGTAA 648

* ****************************** **************************

TMV2 TTAGATTTAAAAATGTTATAATTCATAATTGACATTATTACGTGTTTGTTTTAGATATTA 720

Rysto TTAGATTTAAAAATGTTATAATTCATAATTGACATTATTATGTGTTTGTTTTAGATATTA 708

**************************************** *******************

TMV2 ATCTATGACAAGTCTTTGCTTTTCTATTTATTTACTTTGAGATTATTTTCCACTGTAATG 780

Rysto GTCTATGACAAGTCTTTGCTTTTCTATTTATTTACTTTGAGATTATTTTCCACTGTAACG 768

********************************************************* *

TMV2 TAGGGATGAGGCTGAGTGCGTCAAGAAACTAGTTGATGACATATTTCCTAAGTCACTTCA 840

Rysto TAGGGATGAGGCCAAGTGCGTCAAGAAACTAGTTGATGACATATTTCCTAAGTCACTTCA 828

************ **********************************************

TMV2 AATTATTTCACCTTTCCCGGTAAACTTAGTGGGTATGAAATCTCAGGTTGAGAAAGTAAC 900

Rysto AATTATTTCACCTTTCCCGGAAAGCTTAGTGGGTATGAAATCTCAGGTTGAGAAAGTAAC 888

******************** ** ************************************

TMV2 CTCATTATTAGATATGGAATCAAATGATGTTCGCTCTATCGGAATTTGGGGTATGGGCGG 960

Rysto CTCATTATTAGATATGGAATCAAACGATGTTCGCTCTATTGGTATTTGGGGTATGGGCGG 948

************************ ************** ** *****************

TMV2 CATCGGCAAGACAGAAATTGCAAATATTCTACATCAAAGATACCGCCATCGATTTGAAGC 1020

Rysto CATCGGCAAAACAGAAATTGCAAATGTTCTACATCAAAGATACCGCCATCAATTTGACGC 1008

********* *************** ************************ ****** **

TMV2 TGATTGTTTTCTTGGTGATGTTGGAAAACTTCACCAGAAAAATGGACTAACATGGCTACA 1080

Rysto TGATTGTTTTCTTGGTGATGTTGGAAAACTTCATCAGAAAAATGGACTAACGTGGCTACA 1068

********************************* ***************** ********

TMV2 ACAAGTCGTCATTTGCAAGCTCTTGGGTGAAAAATTGACTCTAACTAGTGAGCATGAAGG 1140

Rysto ACAAGTCGTCATTTGCAAGCTCTTGGGTGAAAAATTGACTCTAACTAGTGAGCATGAAGG 1128

************************************************************

TMV2 GATGAATATTTTAAAGAATATGCTTCGCTGGAAGAAAGTTCTGTTCATCATCGATGATGT 1200

Rysto GATGAATATTTTAAAGAATATGCTTCGCTGGAAGAAAGTTCTGTTCACCATCGATGACGT 1188

*********************************************** ********* **

TMV2 AAACCATCAAGAACAGTTGGAATTTTTGGTTGGAGAGCCAGAGTGGTTTGGTAGGGGTAG 1260

Rysto AAACCATCAAGAACAGTTGGAATTTTTGGTTGGAGAGCCAGAGTGGTTTGGTAGGGGTAG 1248

************************************************************

TMV2 CAGAATTATTTTAACGGCAAGAGACAAGCACCTGTTAATCAGTCACGTTGGGGATAACGT 1320

Rysto CAGAATTATTTTAACAGCAAGAGACAAGCACCTATTAATCAGTCACGTTGGGGATAATGT 1308

*************** ***************** *********************** **

TMV2 GTATGAAGTCCAACTATTATCGGAGGATGAAGCACTTGAATTGTTCAGTAGGCATGCTTT 1380

Rysto GTATGAAGTCCAACTATTATCTGAGAATGAAGCACTTGAATTGTTCAGTAGACATGCTTT 1368

********************* *** ************************* ********

TMV2 TAGAGAAAAATCACCAAAAGAAGATTTTTTGGAACTTTCGAGCGAAGTGGTGGAGCATGC 1440

Rysto TAGAGAAAGATCACCAAAAGAAGATTTTATGGAACTTTCAAGACAAGTGGTGAAGCATGC 1428

******** ******************* ********** ** ******** *******

TMV2 TGGGGGACTCCCTTTAGCTCTTAAAGTTTTGGGTTCTTCATTTTACGGACGAGACAAAAA 1500

Rysto TGGTGGACTCCCTTTAGCTCTTAAAGTTTTGGGTTCTTCATTTTACGGACGAGACAAAAA 1488

*** ********************************************************

TMV2 GCACTGGAGACACATAATTGATCGGCTGAAGAGAATCCCTCACAAGGATATTCTAGGAAA 1560

Rysto GCACTGGAGACACATAATTGATCGGCTGAAGAGAATCCCTCACAAGGATATTCTAGGAAA 1548

************************************************************

TMV2 GCTTAGGCTTAGTTTTGATTGTCTGGACAAAGATGAGAAGGAATTATTTCTAGATATTGT 1620

Rysto GCTTAGGCTTAGTTTTGATGGTCTGGACAAAGATGAGAAGGAATTATTTCTGGATATTGT 1608

******************* ******************************* ********

TMV2 ATTTCTTTATATTGCACGCTTGAGTAGCTATGATTTTGATATTTGTGTGGAACAAGTACA 1680

Rysto ATTTCTAGAGATTGCATGCTTGAGTGGATATGATTTTAATATTTATGTGGAACAAGTACA 1668

****** * ****** ******** * ********* ****** ***************

TMV2 GAGATATGTGAGTCGTGGCTTTCTAATAGATTACCTTATCGAAAAATCTCTGTTATCCAA 1740

Rysto GAGATATGTGAGTCGTGGTTTACTAATTTATTACCTTATTGAAAAATCTCTGTTATCCAT 1728

****************** ** ***** ********** *******************

TMV2 CGACCTGAATAATAGTATTGTGATGCATAATATGATAAGAGAAATGGGAGAAAATGTCAT 1800

Rysto CGACTGGAGTAATAGTATTGTGATTCATAATATGATAAGAGAAATGGGAGAAAATGTCAT 1788

**** ** *************** ***********************************

TMV2 ACGGGAAGAGTACGCTAACAGCAGAATATGGCTTCCCGAGGAGGTTTGTGACCTTTTTAA 1860

Rysto ACGGGAAGAGTACGCTAACAGCAGAATATGGCTTCCCGAGGAGATTTGTGATCTTTTTAA 1848

******************************************* ******* ********

TMV2 AGGGAAGTTGGTAAGCAATATTTAACGTAATTAATAATTTCATAAGCTCTTAGTTCTGTT 1920

Rysto AGGGAAGTTGGTAAGCAATATTTAACGTAATTAATAATTTCATAAGCTCTTAGTTCTGTT 1908

************************************************************

TMV2 AATTTATTTTGTTTCATTTTTATTTGTTAGCCATTAAATATTGTTTCATTTTTTACAGAT 1980

Rysto AATTTATTTTGTTTCATTTTTATTTGTTAGTCATTAAATATTGTTTCATTTTTTACAGAT 1968

****************************** *****************************

TMV2 AACAGAAAAGGTGGAAAGCCTATGTATCCCAAAAGAGTACTATTTTGATGATGATTTTGT 2040

Rysto AACAGAAAAGGTGGAAAGCCTATGTATTCCAAAAGAGTACTATTTTGAAGATGATCTTGT 2028

*************************** ******************** ****** ****

TMV2 TGATTATGGCAATATTTTTAAGAGGATGCAAAGCTTACAAATACTCATAGTTGGTAATGG 2100

Rysto CAATTATAGCAATATTTTCAAGAGGATGCAAAGCTTAAAAACACTCATAGTTGGTGATGG 2088

***** ********** ****************** *** ************* ****

TMV2 AACTTTTAGCTCAAACTGCGCTATCACTTATCTTCCTTCCAGCCTGCGGTTTATTGATTG 2160

Rysto AACTTTTAGCACAAACTGCACTATCACTTATCTTCCTTCCAGCCTGCGGTTCATTGATTG 2148

********** ******** ******************************* ********

TMV2 GTCAGGGTATCCTTCAATTTCATTGCCAGAGAGCTTTGAACCATCACAGCTCGCTATGCT 2220

Rysto GAAAGGGTATCCTTCAATTTCATTGCCAGAGAGCTTTGAACCATCACAGCTTGTGGTGCT 2208

* ************************************************ * ****

TMV2 TTGTTTATGTGAAAGTAGGCTTGTTGAACTTTGGGCAATATCAAAGGTAATACTCTCTTA 2280

Rysto TTGTTTATATAAAAGTAGGCTTGTTGAACTTTGGCCAATATCAAAGGTAACACTCTCTTA 2268

******** * *********************** *************** *********

TMV2 CAAATATTAGACATCAACTGGAGTTGTTTGCAAAGTTCTTGTACAGAATAATGGAATAAT 2340

Rysto CAAATATTAGACATCAACTGGAGTTGT-TGCAAAGTTCTTGTACAGAATAATGGAATAAT 2327

*************************** ********************************

TMV2 AATTCTGAACAAATAGTTGTAATTATAGAATATCATGTATATAATGAGGTTGAAATAAAT 2400

Rysto AATTCTGAACAAATAGTTGTAATTATAGAATATCATGTATATAATGAGGTTGAAATAAAT 2387

************************************************************

TMV2 GTTTTGATAGGCCAATGCATGTAAATTAATCCATCCATGATTTAAATGCTACTTTAAATA 2460

Rysto GTTTTGATAGGCCAATGCATGTAAATTAATCCATCCATGATTTAAATGCTACTTTAAATA 2447

************************************************************

TMV2 AATGTTCTGATATACACTATTAACACCAAATTTTTATTTATTTTTGGTTATCAGTCACGG 2520

Rysto AATGTTCTGATATACACAATTAACACCAAATTTTTATTTATTTTTGGTTATCAGTCACGG 2507

***************** ******************************************

TMV2 CATAGGC-CCATAAATATTTTTAATTTTCCAATATTTTAAAAAATTAAATCTTATAAAAA 2579

Rysto CATAAGGCCCATAAATATTTTTACTTTTCCAATATTTTAAAAAATTAAATCTTATAAAAA 2567

**** * *************** ************************************

TMV2 GTTACGATATTAAAGAGGTTTTGTTTTCATTATGAAATTAAAAGAAAAGAAATATATATT 2639

Rysto GTTACGATATTAAAGAGGTTTTG-TTTCATTATGAAATTAAAAGAAAAGAAATATATATT 2626

*********************** ************************************

TMV2 TGGATTTTAAGACAATTATGTATCTCAAAAGTTCTTTTCCGGATTAGCTACCGCCTTTAG 2699

Rysto TGGATTTTAAGACAATTATGTATCTCAAAAGTTCTTTTCCGGATTAGCTACCGCCTTTAG 2686

************************************************************

TMV2 TGATCATAACAACCTTATCTTCTCTTTATATTTTTTCCTACAATATAAAG---------- 2749

Rysto TGATCATAACAACCTTATCTTCTCTTTATCTTTTTTTCCTACAAAATATTTCTATTAGGA 2746

***************************** ****** * * * *

TMV2 ----TTCTTAAGCTC-------ATTGTCTTTTTTTTTTTTTTTTGGAAACAGAAATTGAG 2798

Rysto GTACTACTTAAGCTCACTGTGTTTTTTTTTTTTTTTTTTTNTTTNGAAACAGAAATTGAG 2806

* ********* ** * ************ *** ***************

TMV2 CAACTTGAAGCATTTGGATCTCATGGGCAGCTGTGAGTTAAGAAAAACCCCTAATTTTGG 2858

Rysto CAACTTGAAGCATTTGGATCTCATGGACAGCTGTGAGTTAAGAAAAACCCCTAATTTTGG 2866

************************** *********************************

TMV2 TGATATGCCAAACTTGGAGAAACTATATTTAAGCGGGTGTGTGAATTTGGAAGAGGTCCA 2918

Rysto TGATATGCCAAACTTGGAGACACTAATTTTACACGGGTGTGTGAATTTGGAAGAGGTCCA 2926

******************** **** **** ***************************

TMV2 TCCCTCTCTTGGACATTG-----CAGAATGCTTACTTCTTTGAGTTTGTCTGATTGTCAC 2973

Rysto TCCCTCTCTTGGACAGTGCAGTGCAGAGTGCTTACTTATTTGAGTTTGGAAGGTTGTCGC 2986

*************** ** **** ********* ********** * ***** *

TMV2 AAACTTCAGAAGCTTCCAAAATTTGTCTGCATGGATTCTCTTGAGGATCTCGATCTCAGT 3033

Rysto AAACTTAAGAAGCTTCCAAAATTTGTCTGCGTGGAATCTCTTGAGACTCTCAATCTCCTT 3046

****** *********************** **** ********* **** ***** *

TMV2 GAATGCACAAGGTTAGAAGAATTTCCAGAAATCTGTGGAGATATGCATGGCTTATCAATA 3093

Rysto GAATGCACAAGCTTACAAGAATTTCCAGAAATCTGTGGAGATATGCATCGCTTATCGACG 3106

*********** *** ******************************** ******* *

TMV2 CTCTATCTAGGATCCCCGTGGATAAGAAGCTTACCTCCATCTTTCAGCAGCCTTAGAAAT 3153

Rysto CTCGATGTAGGATCCCCCTGGATAAGAAGCTTACCCCCATCTCTCAGCGGCCTTAGATAT 3166

*** ** ********** ***************** ****** ***** ******** **

TMV2 TTGCAATTGACTGACTGTGAAGTTCTTGAAAGTATTCCGGACGCCATTCAAAATCTTAGA 3213

Rysto TTGCAATTGACTGAGTGTGCAGTTCTTGAAAGTATTCCGGACGCCATTCAAAATCTTAGA 3226

************** **** ****************************************

TMV2 TATCTCAGTATTTCAGGTTGCAATAAACTTGCAACACTGCCAAACAACCTCTTTGAATCA 3273

Rysto TATCTCAGTATTTTAGATTGCAATAAACTTGCAACACTGCCAAACAGCCTCTTTGAATCA 3286

************* ** ***************************** *************

TMV2 CAGCAATTGGAATATCTTTTAATATGGCAATGCTCTGGATTGGTAAAGCTCCCCATATCT 3333

Rysto CAGCAATTGGAATATCTTTTAATACACCGATGTTCTGGATTGGTAAAGCTCCC-CTTTCT 3345

************************ * *** ******************** * ***

TMV2 CTTGGAGTTCAAAAGATTCTCCGTTGGTTAGATATAGATGGATGTGAGAACTTAAAGAAG 3393

Rysto CTTGGAGTTCAAAAGATTCTCCGTTGGTTAAGTATAGATGGATGTGAGAACTTA-AGAAG 3404

****************************** ********************** *****

TMV2 CTTCCAAGCTCGATTCAGATGAAATCCCTTCAAAAGCTCGAGATAGCTAATTCCCCAAAA 3453

Rysto CTTCCAAGCTCGATTCAGATGAAATCCCTTCAAAGTCTCTGGATATCTGATTCCCCAAAA 3464

********************************** *** **** ** ***********

TMV2 TTAGATACATTTCCAGAAATCAATGGAGATATGCATTGCTTGAAAGAACTGACTCTGAAT 3513

Rysto TTAGACACATTTCCAGAAATCAATGGAGATATGCATTACTTGGAAATACTGTCTCTGAAG 3524

***** ******************************* **** ** **** *******

TMV2 TCTACTGGGATAAGAGAAGTGCCTTCGTCCATTGGGAATCTGAGCGGCCTCACTGAGCTA 3573

Rysto TCTACTGGGATAAGAGAACTGCCTTCATCCATTGGGAATCTGAGCGGCCTCACTGATCTA 3584

****************** ******* ***************************** ***

TMV2 AATCTTACAGGTTGTGAAGATCTTCTAAGTCTACCAGACAGCCTCTGCAATTTGATGAAA 3633

Rysto AGTCTTACAGGTTGTGAAGATCTTCTAAGTCTACCAGACAGCCTCTGCAATTTGATGAAA 3644

* **********************************************************

TMV2 CTTCAAAGTCTTTACCTCGACGGGTGCAAAAAGCTAGAGAAGCTTCCAGAAAACATTGGT 3693

Rysto CTTCGAAGTCTTTACCTCGACGGGTGCAAAAAGCTAGAGAAGCTTCCAGAAAACATTGGT 3704

**** *******************************************************

TMV2 GATTTGCAAGATTTACATATACTTGATGCGAGCGATACTGCAATCTCCCAACCACCTTCC 3753

Rysto GATTTGCAAGATTTACATAAACTTGATGCGAGCGATACTGCAATCTCCCAACCA-CTTCC 3763

******************* ********************************** *****

TMV2 TCCATCACCAAGCTTGGCAAACTGTGGAAGTTACGATTCTCACATGAAAAACAACTTCAA 3813

Rysto TCCATCACTAAGCTTGGCAAACTGTGGAGGTTACGATTCTCACATGAAAAACAACTTCAA 3823

******** ******************* *******************************

TMV2 TATTCCTCAAGTTTTGTCTTGAATCAAGTATCAGGTTTATCGTCCTTGACATCACTTGAT 3873

Rysto TATTCCTCAAGTTTTGTCTTGAATCAAGTATCAGGTTTATCGTCCTTGACATCACTTGAT 3883

************************************************************

TMV2 CTTAATAATCACAACATATTGAGTGGACTTCCTGAGGATTTAGGATCTTTGCAGTCTTTG 3933

Rysto CTTAATAATCACAACATATTGAGTGGACTTCCTGAGGATTTAGGATCTTTGCAGTCTTTG 3943

************************************************************

TMV2 AAAAAACTGAGTCTAAGTGGAAGCAATATTTCTTGTTTACCAAAAAGCTTCAAAGGACTC 3993

Rysto GAAAAACTGAGTCTAAGTGGAAGCAATATTTCTTGTTTACCAAAAAGCTTCAAAGGACTC 4003

***********************************************************

TMV2 TTACACCTTCAGCATCTAAACGTACAATTCTGTCAGAATCTTAATAAATTGCCCGGAGAG 4053

Rysto TTACACCTTCAGCATCTGA-TGTACCCTTCTGTCAGAATCTTAATAAATTGCCCGGAGAG 4062

***************** * **** *********************************

TMV2 CTACCCCCAAATTTAAAGGAGCTATGTGCAGATTATCATTTAGCCTTAATGAGCATCAGA 4113

Rysto CTACCCCCAAATTTAAAGGAGCTATGTGTAGATTATCATTTAGCCTTAATGAGCATCAGA 4122

**************************** *******************************

TMV2 GATCTCGTAATTCATTCTCCTAAGCTGTGTAGGCTCGTGATATCCGACTGTGGAGCCGTC 4173

Rysto GATCTCGTAATTCATTATCCTAAGCTGTGTAGGCTTGGGATATCCGACTGTGGAGCCGGT 4182

**************** ****************** * ********************

TMV2 TCAAGTGAACAAGTTAATGTGTTCCTACAATATTTTCTCATGACATGCATCCAGGTTTTT 4233

Rysto CTCAAGTGAACAAGTTAATGTGTCCTACAATATTTTATCAGGACATGCATCCAGGTTTTT 4242

* * * * * ************** *** *******************

TMV2 ATCTTATATATGAAATGTTGAACATTCTCGTCAAATTGATTGATTTCTAGTAGTAATAAT 4293

Rysto ATCTTATATATGAAATGTTGAATATTCTCGTCAAATTGATTGATTTCTAGTAGTAATAAT 4302

********************** *************************************

TMV2 AATTTCTATTCTATTGCATGCAGTTTGACTTTCTCCAAAGAGATTATTTTCTCATTTTT- 4352

Rysto AATTTCTATTCTATTGCATGCAGTTTGACTTTCTCCAAAGAGATTATTTTCTCATTTTTT 4362

***********************************************************

TMV2 TTTCCTGATCAAGTCGGAATTCCAGAGTTGTTTGATTATCATCGGTTTATAAATCAAAAA 4412

Rysto TTNCCTGATCAAGTCAGAATTTCAGAGTTGTTTGATTATGATCGGTTTACAAATCAAAAA 4422

** ************ ***** ***************** ********* **********

TMV2 GTGATCGCAATTGATCTGAACCCATCTTGGTATACCGATAAATTCATGGGTTTTTGGATA 4472

Rysto GAGATGTCAATTGATCTGAACCCATCTTGGTATACCGATAAATTCATGGGTTTTTGGATA 4482

* *** *****************************************************

TMV2 AGCCATGGTCCTACTACACTGAACTACACAAGCTTAGAAGCTACATTGGTCTGCAAATCT 4532

Rysto AGTTATGGTCTTACTACACTGAACTACACAAGATTAGAAGCTACATTGGTCTGCAAATCT 4542

** ****** ********************* ***************************

TMV2 GACCCTGAAAGAAAATATTCCTTGAAGTATAACAACTTTGGACAATTGTATATCGAGTCT 4592

Rysto GACCCTGAAAGAAAATATTCCTTGAAGTATAACTACTTTGGACAATTGTGTA-CGAGTCT 4601

********************************* *************** ** *******

TMV2 CCTTCCATTTGTTGCTTCTACATACCATTTGAAACATTGTGGAATAATGGTTATGGCAAT 4652

Rysto CCTTCCATTTGTTGCTTCTACATACCATTTGAAACACTGTGGAAT---GCTTCTGGCAAT 4658

************************************ ******** * ** *******

TMV2 AAAGAAGGGAAGAATCCAAATGATTATTACATGTTGGAGGTATATGTTTTGTACAATAAG 4712

Rysto AAAGAAGGGAAGAATCCAAATGATTATTACATGTTGGAGGTATCTAATAGGTACAGTCTG 4718

******************************************* * * ***** * *

TMV2 AGGGAAGAACTACGCTGCTGGGGAATTCGCCTGGAGTATGAAAAGGAGGAGGCAATGAGT 4772

Rysto GAG---AAAGAACGATGCTGGGGAATTCGCCTGGAGTATGAAAAGGAGGAGGCAATGAGT 4775

* ** *** *********************************************

TMV2 GATACTGGTCGTCCAAAAAAGAAAAGGAAGCAATGAGTGATGCAAAGGTCTGTTTATGAC 4832

Rysto GATACTGGTCGTCCAAAAAAGAAAAGGAAGCAATGAGTGATGCAAAGGTCCTTGTTTTAC 4835

************************************************** * * * **

TMV2 TTGTTTTACATCACAATATTTAAATGTTTTTTATTTCTTTCTTAAAACTCCGTATTCAAC 4892

Rysto ATCACAA---------TATTTAAAT----------------------------------- 4851

* *********

TMV2 CAAACACGTCTCATAAAATTGTATGGAAAGAATATGTTCTATATTGTTGATACAATTTGT 4952

Rysto ------------------------------------------------------------ 4851

TMV2 ATATGAGGGGTTTGTATCGATGATACATGGATGAAATTTTTATTTATAATGTTGGTTTTT 5012

Rysto ------------------------------------------------------------ 4851

TMV2 GAGTTTGAAACAACTTCTTTACACTTATAA---GGTAGGTACGTTGAGTATCC 5062

Rysto ------------------------TTATAATAAGGTAGATACGTTGAGTATCC 4847

**Fig. S10. DNA sequence comparison of the *TMV2* genomic region of ‘White Lady’ and the *Ry_sto_* region of dH ‘Alicja’** published by Grech-Baran et al. [42] in Supplementary Fig. 7. Start and stop codons are in red. Primers used to clone *Ry_sto_* as a candidate gene in c630 are marked with red boxes. Position of primer sequences used to detect *TMV2* and *Ry_sto_* specific expressions are highlighted by green and yellow boxes, respectively. The alignment was generated using the web tool Clustal Omega.
