# Supplementary material for: Mapping and DNA sequence characterisation of the Rysto locus conferring extreme virus resistance to potato cultivar ‘White Lady’
Source: PLoS One. 2020 Mar 31;15(3):e0224534. doi: 10.1371/journal.pone.0224534 (PMC7108733; doi:10.1371/journal.pone.0224534)
Supplement: S11 Fig — (DOCX) [file pone.0224534.s012.docx]

**Fig. S11. Detection of transgenes in putative transgenic Désirée lines generated by the constructs P1-2, P3-4, DR and T3** (original gel photos)**.** WL, ‘White Lady’; D, ‘Désirée’. Independent transgenic lines are numbered. Numbers in red indicate the lines considered to be transgenic. PCR fragments were generated from genomic DNA with the primer pairs presented in Table 1. Two bands located close to each other were expected using the Phloem1.spec primer pair in transgenic D lines. By contrast, only one band with different mobility resembling WL or D was detected in plants transformed with the P1-2 construct. A potential explanation is the lack of the 100% identity of primer sequences with the corresponding D sequence. Thus, in the presence of the WL fragment only the WL specific region is amplified by PCR.
