# Supplementary figures and images for: Mapping and DNA sequence characterisation of the Rysto locus conferring extreme virus resistance to potato cultivar ‘White Lady’
Source: PLoS One. 2020 Mar 31;15(3):e0224534. doi: 10.1371/journal.pone.0224534 (PMC7108733; doi:10.1371/journal.pone.0224534)

**Fig. 5.**


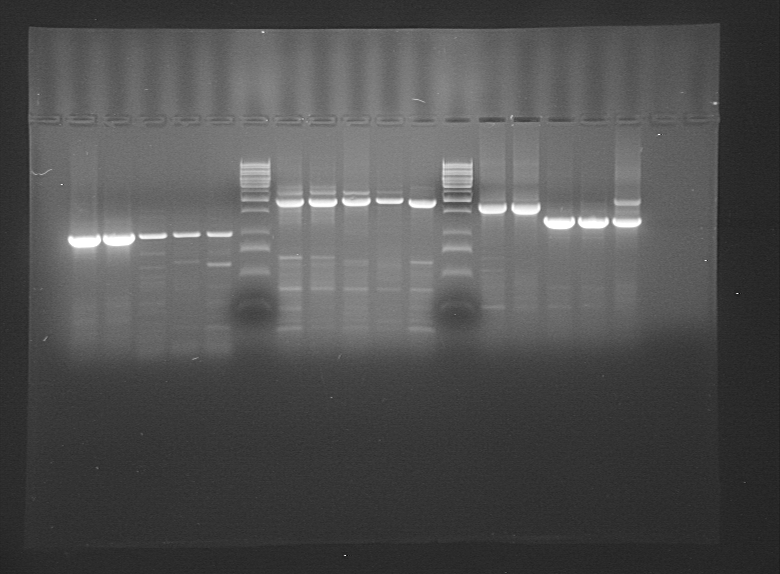


TMV3

WL WL D D S

Phloem 1

WL WL D D S

WL WL D D S +

Disres


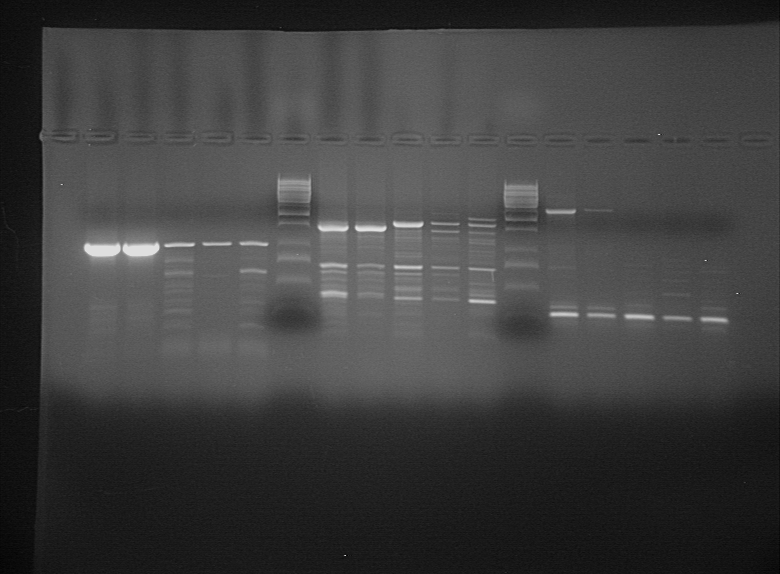


Phloem4


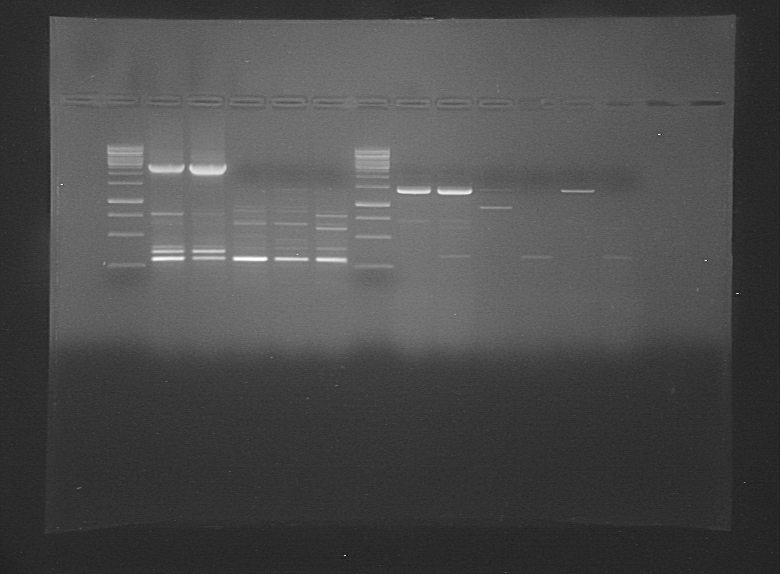


WL WL D D S

**Fig. 6**

1 2 3 4 WL D

TMV2

1 2 3 4 WL D

PVYNTN

Supplement: S1 File — (DOCX) [file pone.0224534.s013.docx]
